# Musculoskeletal Echinococcus infection as a rare first presentation of hydatid disease: case report

**DOI:** 10.1186/s13037-017-0136-y

**Published:** 2017-07-17

**Authors:** A. Marzouki, A. Naam, S. Abdulrazak, B. Soumaré, K. Lahrach, F. Boutayeb

**Affiliations:** 1 Department of Trauma and Orthopedic Surgery A Hassan II Teaching Hospital, Faculty of Medicine and Pharmacy Sidi Mohammed Ben Abdellah University, Fes, Morocco; 2 CHU Hassan II, Faculté de Médicine et de Pharmacie, Université Sidi Mohammed Ben Abdellah, Centre Hospitalier Hrazem, BP : 1835 Atlas, Avenue Hassan II, Fès, Morocco

**Keywords:** Primary, Hydatidosis, Subcutaneous, Elbow, Albendazole

## Abstract

**Background:**

Hydatid disease (HD) is a cosmopolitan parasitosis caused by *Echinococcus granulosus* that can potentially affect any part of the human body. Liver and lungs are the most frequent localizations. Primary musculoskeletal hydatidosis are seldom reported in literature and their incidence is unknown.

**Case presentation:**

We deem interesting to report a case of a primary hydatid cyst in a 25-year-old patient who presented with a painful swelling of the right elbow. Meticulous examination and preoperative imaging did not reveal other localizations. Patient was admitted for surgery where total excision of cyst was done without intraoperative spillage and a cutaneous skin flap was subsequently required to make up for soft tissue loss.

**Conclusion:**

Primary hydatid cysts are sometimes difficult to diagnose preoperatively. It should be considered in the differential diagnosis of subcutaneous cystic lesions in regions where hydatid cysts are endemic. Currently, surgical excision is deemed first choice treatment for solitary cysts and antihelminthic treatment should be initiated preoperatively in the case of risk of rupture or in the event of complications.

## Background

Hydatid disease is an anthropozoonosis caused by Echinococcus species. Species mainly involved include *granulosus, multilocularis* and *oligarthrus,* with *granulosus* commonly responsible for cysts in predators such as dogs, wolves, and foxes as well as intermediate hosts such as sheep, goats, and cattle [1]. Humans are a coincidental intermediate host. The disease is more frequent in the Middle East, Central Europe, Australia, South America and the Mediterranean basin, where livestock breeding is very rampant. Several strains of *granulosus* appears to be commonly found in North America, Morocco, Tunisia, Kenya, Kazakhstan, western China and Argentina.

Parasite larvae can develop in any part of the human body with the liver (68.8–80%) and lungs (10–22.4%), representing the most frequent localizations. Other rare localizations reported in literature include the spleen, peritoneum, skeleton, kidney, brain, cardiac muscle and even sub cutis [2, 3]. Subcutaneous hydatid cysts are secondary in nature, resulting from migration of larvae from a primary site after spontaneous or trauma-induced cyst rupture or could even be iatrogenic after release of parasite material during invasive treatment procedures. Primary musculoskeletal hydatid cysts are rare not to mention subcutaneous elbow hydatid cysts even in countries endemic to *Echinococcus.*

We hereby report a case of primary subcutaneous hydatid cyst of the elbow in a 25-year-old patient with a 3-year history of an incipient right elbow swelling.

The authors intend to highlight the pitfalls in the diagnosis and management of primary musculoskeletal hydatid cysts through a rare case report and a review of relevant literature.

## Case presentation

A 25-year-old man, Middle Eastern origin, history of exposure to livestock was admitted to our unit complaining of a painful swelling of the right elbow. The swelling developed over 3 years and was associated with recent pain without fever or rigors, and pruritus. Anamnesis did not reveal any trauma to the elbow or prior medication.

**Fig. 1:**
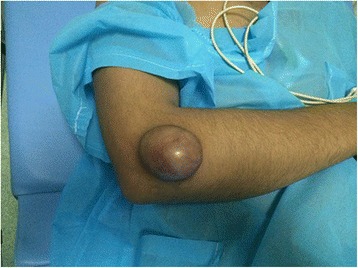
Right lateral elbow mass with distension of overlying skin

He was afebrile on admission with good general conditions, and physical examination revealed a tender right lateral elbow mass with distension of overlying skin. Mass measured 4 cm by 6 cm and there were no signs of excoriations nor fistula (Fig. 1).

On day 3, patient became febrile with a temperature of 38.8 °C. Lab test demonstrated a normal total WBC count of 5.0 × 10^9^ cells/L, eosinophil level of 158 cells/L, and a normal erythrocyte sedimentation rate of 15 mm/h. Liver function tests were unremarkable. Hydatid serology was negative and there was no modification in the appearance of the mass.

**Fig. 2:**
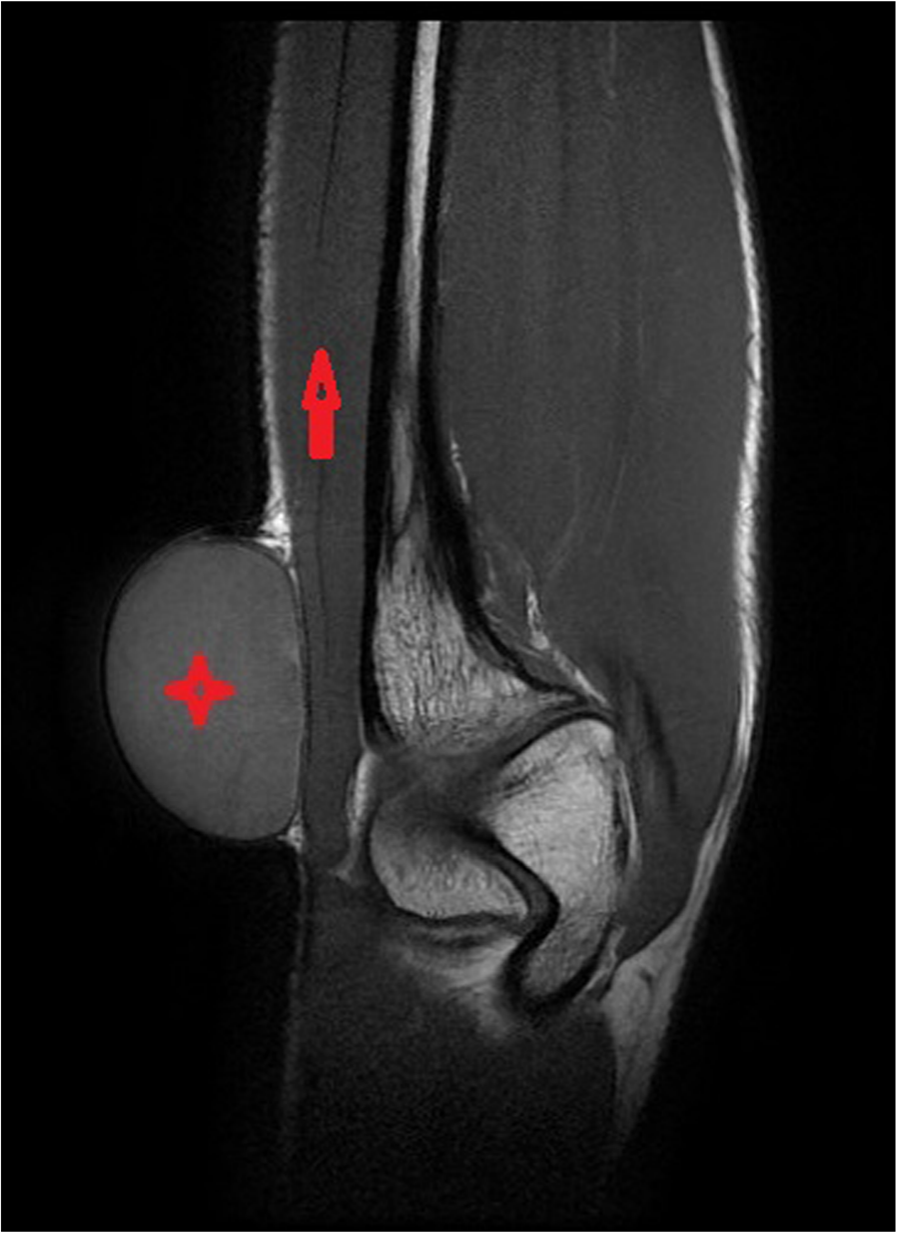
MRI image; T1 coronal view showing cystic lesion with fine capsule () in close relation with flexor carpi ulnaris ()

**Fig. 3:**
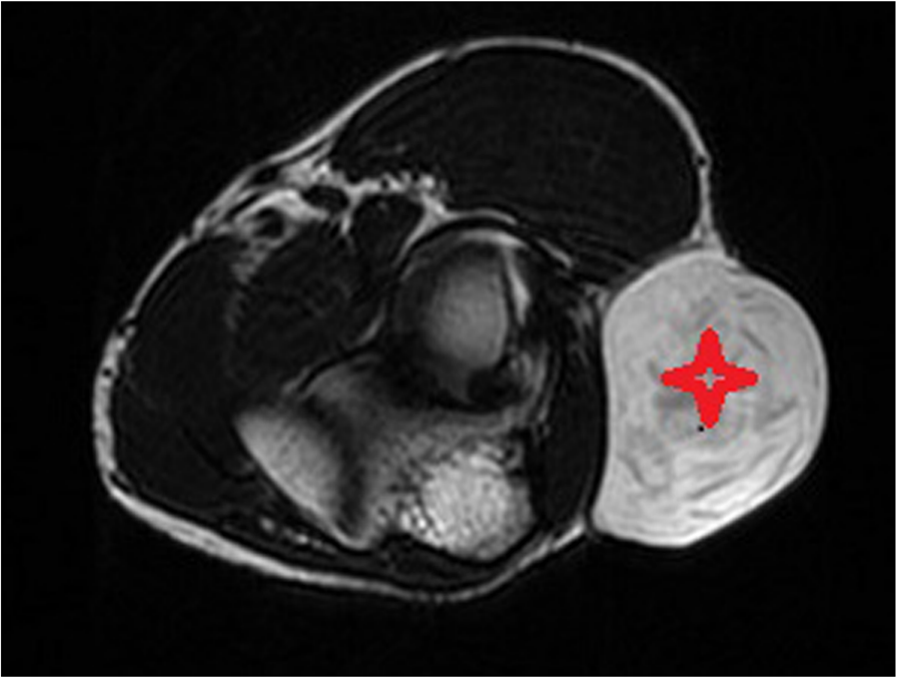
MRI image; T2 axial view showing unilocular cyst with thin septations

Plain elbow and chest radiographs were also unremarkable albeit diffuse soft tissue swelling of the elbow: there were no bone erosions nor calcifications. Ultrasound came back for a cystic lesion of the elbow with several floating membranes without color Doppler test. Magnetic resonance Imaging (MRI) depicted a unilocular cyst with multiple septations giving it a multivesicular or rosette appearance, confined to the soft tissues, adjacent to the medial elbow muscles without infiltrating bone nor surrounding neurovascular structures (Figs. 2 and 3).

**Fig. 4:**
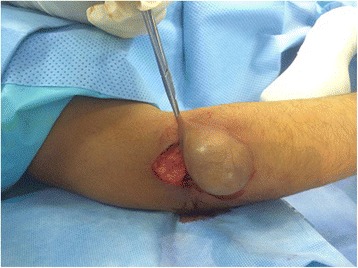
Peroperative image showing careful pericystectomy

**Fig. 5:**
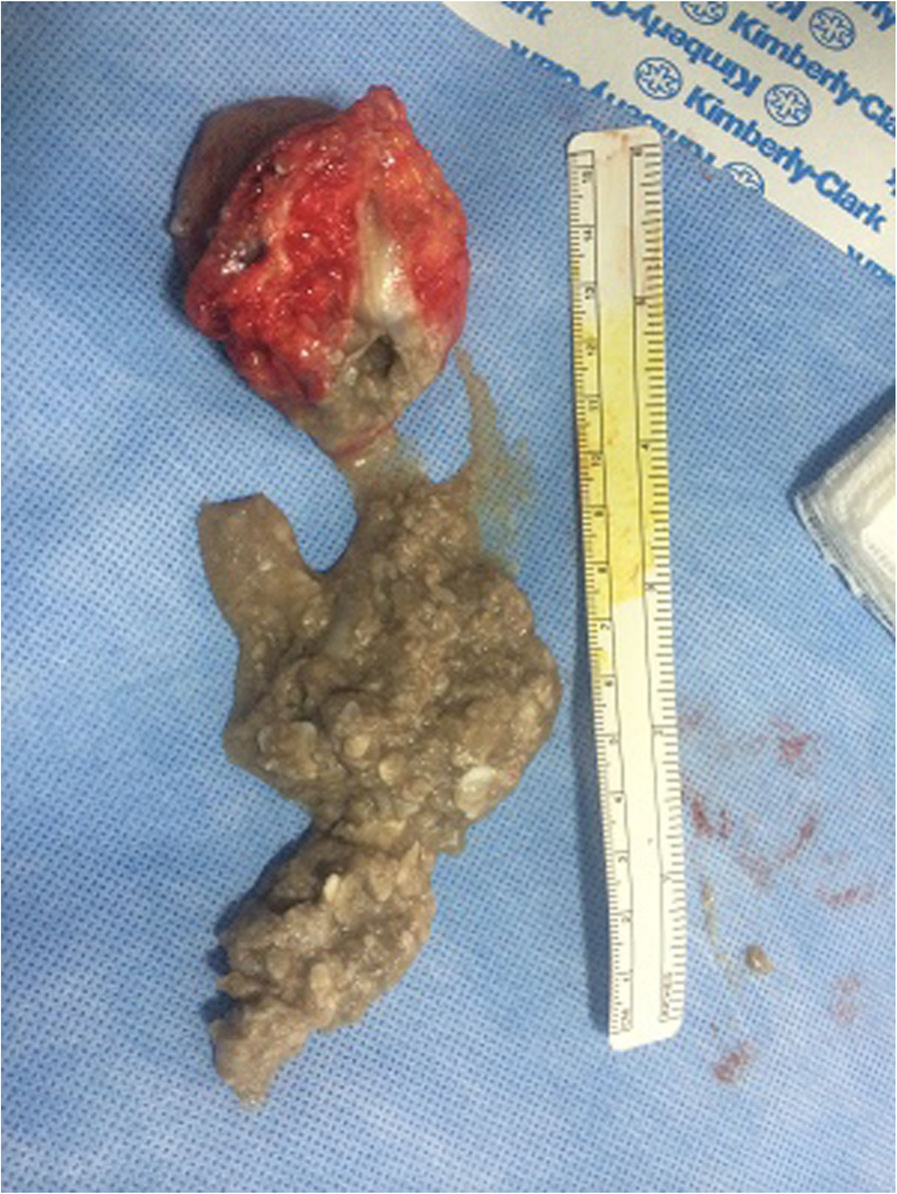
Surgical specimen with hydatid sand after opening of cyst

**Fig. 6:**
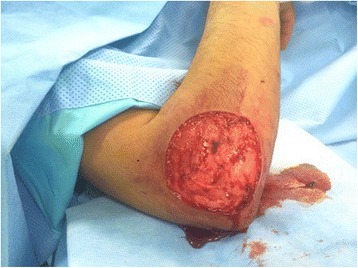
Surgical wound with extensive soft tissue loss

Patient was prepared for elective surgery with consent and antihelminthic therapy was initiated preoperatively for 5 days. En block surgical excision of the mass under general anesthesia was undertaken. Care was taken to remove the mass en block without perforating the cyst wall, through meticulous pericystectomy along surrounding muscle fibers (Fig. 4). The cyst was multivesicular containing daughter cysts and were filled with muddy substance typical of hydatid disease (Fig. 5). After excision, extensive washout of the surgical field was carried out. Wound could not be closed due to massive soft tissue loss (Fig. 6).

The operating field was covered with adequate dressing. Cutaneous skin flap was done subsequently with favorable outcome and no signs of local recurrence 2 years after surgery.

## Discussion

Hydatid disease is an anthropozoonosis caused by Echinococcus species; humans represent intermediate hosts in parasite life cycle when they occasionally ingest eggs through contaminated food or water [2, 3]. The shell of parasite eggs opens in the presence of upper gastrointestinal tract acidity. Oncospheres, so released in small bowel, penetrate the intestinal wall, and are carried out through portal circulation into the liver. Having made it pass the portal filter, they could potentially reach every organ with the lungs serving a secondary filter. A possible dissemination through lymphatic channels has also been reported [4, 5]. The majority of the hydatid cysts occur in the liver, the lungs, or both, but muscular and subcutaneous area is considered an unsuitable site for infestation.

Thus primary soft tissue involvement is very rare; causing a diagnostic challenge [6]. It is widely postulated that the low prevalence of this form of the disease is potentially due to the physical barriers to the hematogenous dissemination of cysts created by hepatic sinusoids and pulmonary capillaries. In addition, it is widely believed that the higher lactic acid concentration in skeletal muscle and mechanical factors, such as contractions make encystment less likely. The most common musculoskeletal sites include pelvic, thigh, and paravertebral musculature [7]. Another compatible hypothesis is one of a spontaneous resolution of primitive hepatic localization but with systemic diffusion of the parasite and positivity of serological exams [8–10].

Cysts may present in several ways, including a slow-growing lump with variable pain or sudden onset of symptoms due to cyst rupture. Rupture of cysts releases antigens into muscles thus causing an inflammatory response that may be complicated by secondary bacterial infection [11]. More so, involvement of adjacent structures, such as blood vessels, may result in vascular insufficiency [12], whereas muscle infiltration may produce mechanical limitation of movements [13]. Tuna et al. [14] reported a case of upper extremity hydatid cyst associated with peripheral neuropathy in relation to median nerve compression.

Diagnosis is based on clinical evidence such as anamnestic data pertaining to origin and history of exposure to livestock, presentation as well as radiological backing. Hydatid serology is often negative in 1 out of 2 cases of extra hepatic hydatid disease as was the case in our patient. Ultrasonography (USG) is a very useful diagnostic imaging tool for hydatid disease. Not only does it detect liver cysts but it also allows the classification and staging of liver hydatid disease [15, 16]. Nevertheless, we strongly believe the sensitivity of USG for floating membranes, daughter cysts, and hydatid sand in purely cystic lesions make it very ideal in the initial workup of even uncommon localizations. In general, MRI has a higher specificity and sensibility than ultrasound for hepatic hydatid cyst [17]. It also allows a better characterization of anatomical relations and aids surgical management of cyst as was the case in our patient.

It is essential to establish definitive preoperative diagnosis of skeletal muscle hydatid cysts. This contraindicates certain treatment options like marginal excision or incisional biopsy due to the likelihood of dissemination and anaphylactic shock on spillage. Thursky et al. [18] reported a case of cyst rupture complicating the surgical drainage of an infected primary musculoskeletal hydatid cyst after previous fine needle aspiration. Pericystectomy remains treatment of choice in musculoskeletal hydatid cysts. Percutaneous aspiration, infusion of scolicidal agents like chlorhexidine gluconate, and reaspiration (PAIR), under imaging (ultrasound or CT) guidance can be used as alternative to surgery in inoperable cases.Ormeni et al. [19] reported percutaneous puncture with concomitant injection of ethanol and polidocanol in the cyst cavity as treatment for selected cases of primary muscle hydatidosis without recurrence. Regardless, the inherent risk of rupture and hydatid matter dissemination cannot be ruled out completely.

Supplementary chemotherapy with antihelminthics for skeletal muscle hydatid disease is controversial and currently no evidence provides sufficient backing on the benefit of its association with conservative treatment. Albendazole remains the gold standard drug administered in adjuvant therapy. Combination therapy with praziquantel has been reported in the treatment of inoperable hydatidosis [20]. In cases affecting skeletal muscles where surgical excision is possible, the rationale of adjuvant chemotherapy is to reduce the risk of dissemination during surgery and to prevent recurrence [21]. The inherent risk of dissemination of cyst during surgery due to its large size was a key factor in our indication of neoadjuvant therapy with albendazole.

## Conclusion

This case of primary skeletal muscle hydatidosis illustrates the significant morbidity associated with hydatid disease. Preoperative diagnosis is important in the management of hydatid cyst. Pericystectomy potentially combined with neoadjuvant therapy could help reduce complications and recurrence in large hydatid cysts.

## Abbreviations

MRI: Magnetic Resonance Imaging
USG: Ultrasonography
